# The Surface-Exposed Protein SntA Contributes to Complement Evasion in Zoonotic *Streptococcus suis*

**DOI:** 10.3389/fimmu.2018.01063

**Published:** 2018-05-16

**Authors:** Simin Deng, Tong Xu, Qiong Fang, Lei Yu, Jiaqi Zhu, Long Chen, Jiahui Liu, Rui Zhou

**Affiliations:** ^1^State Key Laboratory of Agricultural Microbiology, College of Veterinary Medicine, Huazhong Agricultural University, Wuhan, China; ^2^College of Life Science and Technology, Huazhong Agriculture University, Wuhan, China; ^3^Cooperative Innovation Center of Sustainable Pig Production, Wuhan, China; ^4^International Research Center for Animal Diseases (MOST), Wuhan, China; ^5^Key Laboratory of Preventive Veterinary Medicine in Hubei Province, Wuhan, China

**Keywords:** *Streptococcus suis*, surface protein SntA, C1q, complement evasion, pathogenesis

## Abstract

*Streptococcus suis* is an emerging zoonotic pathogen causing streptococcal toxic shock like syndrome (STSLS), meningitis, septicemia, and even sudden death in human and pigs. Serious septicemia indicates this bacterium can evade the host complement surveillance. In our previous study, a functionally unknown protein SntA of *S. suis* has been identified as a heme-binding protein, and contributes to virulence in pigs. SntA can interact with the host antioxidant protein AOP2 and consequently inhibit its antioxidant activity. In the present study, SntA is identified as a cell wall anchored protein that functions as an important player in *S. suis* complement evasion. The C3 deposition and membrane attack complex (MAC) formation on the surface of *sntA*-deleted mutant strain Δ*sntA* are demonstrated to be significantly higher than the parental strain SC-19 and the complementary strain CΔ*sntA*. The abilities of anti-phagocytosis, survival in blood, and *in vivo* colonization of Δ*sntA* are obviously reduced. SntA can interact with C1q and inhibit hemolytic activity *via* the classical pathway. Complement activation assays reveal that SntA can also directly activate classical and lectin pathways, resulting in complement consumption. These two complement evasion strategies may be crucial for the pathogenesis of this zoonotic pathogen. Concerning that SntA is a bifunctional 2′,3′-cyclic nucleotide 2′-phosphodiesterase/3′-nucleotidase in many species of Gram-positive bacteria, these complement evasion strategies may have common biological significance.

## Introduction

*Streptococcus suis* are recognized as an important swine and human pathogen (1). Among the 33 serotypes, *S. suis* serotype 2 (SS2) is the most virulent and prevalent one, which is also an emerging zoonotic pathogen (2). Two large-scale outbreaks of severe human SS2 infection occur in 1998 and 2005 in China causing 229 infections and 52 deaths (3, 4). In 2005, the streptococcal toxic shock like syndrome (STSLS) is first reported to occur in the human. An early burst of inflammatory cytokines could result in the STSLS with death as quickly as 13 h after SS2 infection, and subsequently SS2 breaks through blood–brain barrier (BBB) to cause disease, particularly meningitis (1, 5). Bacterial pathogens evade host innate immune defenses and maintain a high dose in blood causing bacteremia and septicemia. During these processes, the host complement system is an important factor facilitating clearance of bacterial pathogens (6, 7).

The complement system consists of more than 50 plasma and cell surface proteins. As a first line of defense against pathogenic intruders and a mediator between the innate and adaptive immune response, it plays an essential and efficient role in rapid recognition and elimination for invading pathogens. The complement has three independent but interactive activation pathways: the classical pathway, alternative pathway, and lectin pathway (8). These three different complement pathways are stimulated by different foreign substance through specific recognition molecules (4). All the complement cascades result in the deposition of C3b to amplify the cascades, and mediate phagocytosis and adaptive immune responses by binding to complement receptors; the release of pro-inflammatory anaphylatoxins and chemoattractant C5a and C3a; and formation of membrane attack complex (MAC; C5b-9) then lead to direct lysis of Gram-negative bacteria (9).

Although host complement can rapidly recognized and eliminated foreign microorganisms, it also offers many interference sites that can disrupt this balanced network of protein interactions by complement-binding proteins leading to failure of elimination by host. Complement-binding proteins can be identified from both host and pathogens. These complement evasion mechanisms include (I) recruiting or mimicking of complement regulators; (II) modulating or inhibiting complement by direct interactions; and (III) enzymatic degradation by complement components (4).

C1q is the recognition subunit of C1 complex to trigger the classical complement pathway, following the recognition of IgG or IgM-bearing immune complexes (10). Proteins that interact with C1q have been identified widely in Gram-negative bacteria, such as *Salmonella Minnesota* (11), *Escherichia coli* (12–14), *Klebsiellapneumoniae* (15), *Legionella pneumophila* (16), *Bacillus anthracis* (17), *Moraxella catarrhalis* (12), nontypeable *Haemophilus influenzae* (12), but in Gram-positive bacteria the research is not much, except for Group B *Streptococci* (18, 19), *Streptococcus pyogenes* (20–22) and *Staphylococcus aureus* (23).

In our previous study, surface protein SntA of *S. suis* without any unknown function has been characterized to be a heme-binding protein which involved in the pathogenesis of *S. suis* in pigs. SntA can interact with the host antioxidant protein AOP2 and consequently inhibit its antioxidant activity (24). Complement C1q is identified as another interacting partner of SntA when we screen the SntA binding proteins in the host. In the present study, we demonstrate that SntA is an important player in complement evasion of this important zoonotic pathogen.

## Materials and Methods

### Strains and Culture Conditions

*Streptococcus suis* serotype 2 strain SC-19 (GenBank accession number: NZ_CP020863.1) used in this study was isolated from a sick pig during the epidemic outbreak in Sichuan province of China in 2005. The *S. suis* strains were grown in tryptic soy broth (TSB; Difco, France) or on tryptic soy agar (TSA; Difco) plates supplemented with 5% newborn bovine serum (Sijiqing, Hangzhou, China) at 37°C. The *E. coli* DH5α and BL21 (DE3) strains were grown in LB broth or on LB agar plates at 37°C. The bacterial strains, plasmids, and primers used in this study are listed in Table 1.

**Table 1:** Bacterial strains, plasmids, and primers used in this study.

Strains, plasmids, and primers	Description and sequence (5′ → 3′)	Source, reference, and products
***Escherichia coli* strains**		
DH5α	Host for cloning vector	Kangwei
BL21 (DE3)	Host for expression vector	Kangwei

***S. suis* strains**		
SC-19	*S. suis* serotype 2, the wild type	(25)
Δ*sntA*	The *sntA*-deleted strain; Em^r^	(24)
CΔ*sntA*	Complemented strain of Δ*sntA*; Em^r^, Spc^r^	This study

**Plasmids**		
pET28a	Expression vector to produce His-fused proteins; lacZ, Kana^r^	Novagen
pET28a-SUMO	Expression vector to produce His-and SUMO-fused proteins; lacZ, Kana^r^	(26)
pQE32	Expression vector to produce His-fused proteins; lacZ, Amp^r^	Qiagen
pSET2	*E. coli*-*S. suis* shuttle vector; Spc^r^	(27)
pSET2:*sntA*	pSET2 containing the intact *sntA* gene and its upstream promoter; Spc^r^ Em^r^	This study

**Primers**		
C1qA_F	GCGTCGACGCTGGCCGTATCCCTG	ORF of *c1qA* gene
C1qA_R	CCAAGCTTTCAGGTGGAGGGGAAG	
C1qB_F	AGAGAACAGATTGGTGGATCCCAGGTCTTCTGCATGCCTGGC	ORF of *c1qB* gene
C1qB_R	CTCGAGTGCGGCCGCAAGCTTTCATGCCTCTGCATCTGGGAAG	
C1qC_F	AGAGAACAGATTGGTGGATCCCAGGACAACTTAAGATGCCATGGG	ORF of *c1qC* gene
C1qC_R	CTCGAGTGCGGCCGCAAGCTTCTAGTTAGGGAAGAGCAAGAAGCCG	
CΔ*sntA*_F	AAAACTGCAGCTCGTTTTTCAAACGAACGTTCAG	*sntA* gene and its upstream promoter
CΔ*sntA*_R	GCGGGATCCTTAATGTTCTTTTTTCTTAGTCCATG	
10065_F	ATGAAAGAAATTAACTACACTGGGGA	ORF of B9H01_10065 gene
10065_R	TTATCCGTTGGTCCTTTTCACTGA	
SntA_F	ATGAATTTTCGTTTCAGTAAGTGTGC	ORF of *sntA* gene
SntA_R	TTAATGTTCTTTTTTCTTAGTCCATGC	
10075_F	ATGAAAAGAATAGGATATGTTTTCATAT	ORF of B9H01_10075gene
10075_R	CTACCAAATATAGGTTCTAATAAAACGG	

### Construction of *sntA* Gene Deletion and Complementary Strains

The *sntA*-deleted strain Δ*sntA* was constructed in our previous study (24). To construct *sntA* complementary strain, a DNA fragment containing the entire *sntA* coding sequence and its promoter and terminator was amplified by using primers CΔ*sntA*_F/CΔ*sntA*_R. The amplicon was subsequently cloned into *E. coli*-*S. suis* shuttle vector pSET2, resulting in the recombinant plasmid pSET2:*sntA*. This plasmid was transformed into the Δ*sntA* strain, and the resulting complementary strain CΔ*sntA* was screened on TSA agar plates supplemented with 100 µg/ml spectinomycin (Spc). The Spc-resistant colonies were verified by PCR and reverse transcription (RT)-PCR analyses by using three pairs of primers SntA_F/SntA_R, 10065_F/10065_R, and 10075_F/10075_R (Table 1).

### Experimental Infection of Mice

To detect the virulence of *sntA* in *S. suis*, a total of 24 female 5-week-old specific-pathogen-free (SPF) Kunming mice (8 mice per group) were intraperitoneally infected with 2 × 10^9^ colony forming unit (CFU)/mouse of SC-19 and Δ*sntA*. Physiological saline was served as a negative control. Then the morbidity, mortality, and clinical symptoms such as limping, swollen joints, shivering, and central nervous system failure of all the mice were observed for 5 days. To evaluate the effect of *sntA* on survival in blood and organs colonization, competitive colonization experiment of SC-19 and Δ*sntA* was performed. 5-week-old SPF Kunming mice (7 mice per group) were co-infected with SC-19 and Δ*sntA* at the ratio 1:1 (5 × 10^8^ CFU/mouse). Bacterial counts in blood, brain, and lung were collected at 6, 12, 24, and 48 h post infection (hpi). Colonization of bacteria in various tissues were analyzed by serial diluted and plated brain and lung samples after homogenizing, and blood samples on TSA agar plates with (Em^+^) and without (Em^c^) 100 µg/ml erythromycin (Em) as described previously (28). The number of live bacteria on TSA agar plates with or without Em was calculated as Δ*sntA*, sum of SC-19 and Δ*sntA*, respectively. The number of SC-19 was calculated as ${\text{TSA}}_{{\text{Em}}^{-}}-{\text{TSA}}_{{\text{Em}}^{+}}$.

### Bactericidal Assays

In human blood and serum killing assays, experiments to evaluate the survival rate of *S. suis* were carried out as described previously (29). Overnight cultures of the *S. suis* strains in TSB were diluted in 1:100 and grown to mid-log phase without agitation at 37°C. The cultures were diluted to 5 × 10^7^ CFU/ml with physiological saline. 50 µl diluted cultures was mixed with 450 µl fresh human blood for human blood killing assay and 20 µl diluted cultures were mixed with 180 µl of normal human serum (NHS) and complement heat inactivated serum by incubation at 56°C for 30 min for human serum killing assay. The resulting mixtures were incubated at 37°C for 30 min. Live bacteria were counted by plating the serial diluted samples on TSA agar plates. The percentage of live bacteria was subsequently calculated as (CFU_after incubation_/CFU_in original inoculum_) × 100%.

In PMNs killing assay, PMNs were isolated from heparinized venous blood by human peripheral blood PMN isolation kit (Haoyang, Tianjin, China). Experiment to investigate the survival rate of *S. suis* in PMNs was carried out as described previously (6). PMNs were mixed with bacteria at MOI = 1:10 in RPMI-1640 medium (Hyclone, USA) supplemented with 20% freshly non-immune human serum and incubated at 37°C under 5% CO_2_ for 30 min. The percentage of live bacteria was subsequently calculated as $({\text{CFU}}_{{\text{PMN}}^{+}}{\text{/CFU}}_{{\text{PMN}}^{-}})\times \text{1}00\%$.

In phagocytosis assay, the experiment was carried out by our previous study (30). Briefly, the 1.0 × 10^6^ RAW264.7 cells for each well were pooled into 12-well plates. Then *S. suis* strains in mid-log phase were added to the plates (MOI = 10:1) and incubated for 30 min at 37°C to allow cells to be phagocytized. After incubation, ampicillin was applied to plates for 1 h to kill the extracellular bacteria. Then, the cells were lysed with sterile distilled water on ice and the bacterial CFU were counted on TSA agar plates. These data are presented as means ± SDs from three separate experiments.

### Complement C3 Deposition and MAC Formation Analysis

C3 deposition and MAC formation assays were performed as previous study (31). Overnight cultures of *S. suis* were diluted in 1:100 in TSB and grown to mid-log phase without agitation at 37°C. Bacteria were collected by centrifugation at 6,000 rpm for 5 min. After two washes, cultures were suspended to 5 × 10^7^ CFU/ml with physiological saline. 300 µl *S. suis* suspensions were mixed with 240 µl freshly undiluted and 1/500 diluted non-immune human serum and incubated at 37°C for 30 min. After two washes, *S. suis* was resuspended in 300 µl anti-C3b monoclonal antibody (20 µg/ml; Abcam, England) to detect C3 deposition and monoclonal anti-C5b-9 antibody (20 µg/ml; Abcam) to detect MAC formation. After incubation for 10 min at room temperature, FITC-conjugate goat-anti-mouse IgG (BD, USA) was used as the second antibody and incubated at room temperature for 10 min. Finally, bacteria were washed and resuspended in 800 µl physiological saline for flow cytometry analysis performed by FACSCalibur (BD). Bacteria were detected by log-forward and log-side scatter dot-plot at middle flow rate. A gating region was set to include the majority of bacteria except debris. 10,000 bacteria/events were acquired and analyzed for fluorescence using log-scale amplifications. C3 deposition and MAC formation capacities were measured by the geometric mean fluorescence intensity (GMFI). GMFI value was measured from three independent experiments performed in duplicate.

### SntA Immunodetection

Western-blot analysis was used to determine the location of SntA expressed in the SC-19, Δ*sntA*, and CΔ*sntA* strains. For cell lysate, cell cultures of *S. suis* strains were collected, resuspended with 1 ml bacterial lysis buffer (0.05 M Tris–HCl, 2.5 mM EDTA, 0.1 M NaCl, 0.25% Triton X-100, pH 8.5~9.0) and boiled 20 min to lysis, the resulting cell lysate were used to prepare SDS-PAGE samples. For cell wall proteins, samples were prepared as described previously (32). Briefly, bacterial cultures were collected; washed once in 50 mM Tris–HCl, pH 7.3; resuspended in 1 ml of osmoprotective buffer (50 mM Tris–HCl, pH 7.3, 20% sucrose, 2.5 µM PMSF) supplemented with 175 U/ml mutanolysin (Sigma-Aldrich, Shanghai, IL, USA); incubated at 37°C for 90 min under constant gentle agitation. After centrifugation at 12,000 *g*, 4°C for 15 min, supernatants containing the cell wall proteins were used to prepare SDS-PAGE samples. For secreted proteins, culture supernatants were collected, filtered (Millipore, 0.22 µm, Shanghai, China), and concentrated to 10 mg/ml, and then the proteins were sedimentated with pre-cooled 10% TCA-acetone solution at 4°C overnight. After centrifugation at 12,000 *g*, 4°C for 15 min, washed sediment with pre-cooled 90% acetone supplemented with 10 mM DTT for three times. The last sediment was resuspended with 8 M urea, 2% CHAPS, 10 mM DTT for next step. For all SDS-PAGE samples immunodetection, total proteins were quantified by BCA protein assay kit (Kangwei, Beijing, China), and then 48 µg cell lysate, secreted, and cell wall proteins were applied to 10% SDS-PAGE. Proteins transferred onto PVDF membrane (Millipore, 0.45 µm). Mouse SntA polyclonal antibody (24) was used for SntA immunodetection, and chemiluminescence detection (Bio-Rad, USA) with HRP-conjugate goat anti-mouse IgG (Antgene, Wuhan, China) by MF-Chemi BIS 3.2 (DNR, Israel).

### Preparation of Recombinant Proteins

Recombinant SntA protein (Protein ID: WP_012027972.1) was expressed and purified as our previous report (24). To obtain the recombinant proteins of the complement C1q subunits, the cDNAs of C1qA, C1qB, and C1qC were amplified from the total cDNA of porcine lung tissue with primers C1qA_F/R, C1qB_F/R, C1qC_F/R, respectively (Table 1). Total cDNA was prepared from total RNA of porcine lung tissue by using RT-PCR. Then the C1qA cDNA was cloned into pQE-32 plasmid (Qiagen, Shanghai, China), while the cDNAs of C1qB and C1qC were cloned into pET-28a-SUMO plasmid (26) to promote soluble expression. The resultant recombinant plasmids were transformed into *E. coli* BL21 (DE3), respectively. The expression of C1qA, C1qB, and C1qC proteins was induced by 1 mM isopropyl-β-d-thiogalactoside for 4 h at 37°C. The recombinant proteins were purified by using Ni-NTA agarose column (Bio-Rad) and quantified by BCA protein assay kit (Kangwei).

### Interaction Between C1q and SntA Proteins

ELISA was used to confirm the interaction between SntA and C1q proteins as described previously (33). For SntA bound assay, the proteins C1q (Quidel, USA), three C1q subunits C1qA, C1qB, C1qC, and BSA were coated to 96-well plates (BIOFIL, Guangzhou, China) with increasing concentrations of 0–7 µg/ml at 4°C for 16 h, respectively. After blocking with blocking buffer (PBS supplemented with 0.05% Tween20 and 1% BSA), 5 µg/ml SntA was poured to each well, and then incubated at 37°C for 1 h. Mouse SntA polyclonal antibody was applied at 37°C for 1 h to test the direct binding capacity. For C1q bound assay, SntA, BSA, and heme were coated to 96-well plates with increasing concentrations of 0–7 µg/ml at 4°C for 16 h, respectively, After blocking with blocking buffer, 5 µg/ml C1q was applied to each well at 37°C for 1 h. Rabbit C1qA polyclonal antibody (Abclonal, Wuhan, China) was applied at 37°C for 1 h to test the direct binding capacity. For inhibition assay, 5 µg/ml human aggregated IgG (Sigma-Aldrich) was coated to plates at 4°C overnight. Human aggregated IgG was prepared by incubating at 63°C for 20 min, immediately placing on ice for 1 h, centrifuging at 16,000 *g* for 5 min and quantification (34). C1q was pre-incubated with increasing concentrations of 0–25 µM SntA, heme, or BSA, and then applied to plates at 37°C for 1 h. Rabbit C1qA polyclonal antibody was applied to plates at 37°C for 1 h to test the indirect binding capacity. All the ELISA assays, the HRP-conjugate goat-anti-rabbit IgG (Antgene), or goat-anti-mouse IgG was used as the second antibody at 37°C for 1 h before chromogenic reaction.

Competitive binding assays were performed as described previously (35). For C1q-IgG binding assay, microtiter plates were pre-coated with 30 µg/ml human C1q overnight at 4°C. After blocking, plates were incubated with the mixtures of 50 µl 2.5 mg/ml human aggregated IgG and 50 µl increasing concentrations of 0–50 µM SntA or BSA in HB^++^ buffer (10 mM Hepes, pH 7.4, 100 mM NaCl, 5 mM CaCl_2_, 1 mM MgCl_2_). After incubation for 1 h at 37°C, plates were washed with washing buffer (HB^++^ buffer supplemented with 0.05% Tween20) and developed with HRP-conjugate goat anti-human IgG Fc (1:4,000; Sigma-Aldrich) at 37°C for 1 h before chromogenic reaction. For C1q–Ag–Ab binding assay, microtiter plates were coated with 2.5 µg/ml tetanus toxoid (Millipore) as an antigen for immune complex formation. After blocking, human tetanus toxin immunoglobulin (1:1,000; Hualan Bio, Xinxiang, China) was applied to the tetanus toxoid-coated plates to form immune complexes for 1 h at 37°C. 50 µl 2 µg/ml human C1q were mixed with 50 µl increasing concentrations of 0–50 µM SntA or BSA. The resulting mixtures were applied to each well at 37°C for 1 h. After washed, polyclonal goat antiserum to human C1q (1:5,000; Quidel, USA) was incubated to plates at 37°C for 1 h. HRP-conjugate rabbit anti-goat IgG (Antgene) was used as the second antibody at 37°C for 1 h before Chromogenic reaction. These data are presented as mean ± SD from three separate experiments.

### Binding of C1q to *S. suis*

Overnight cultures of the *S. suis* strains in TSB were diluted in 1:100 and grown to mid-log phase without agitation at 37°C. The cultures were washed and resuspended in DGHB^++^ buffer to 5 × 10^8^ CFU/ml. *S. suis* suspensions were incubated with 0, 10, and 20 µg/ml C1q proteins at 37°C for 1 h and the total volume was 100 µl. After incubation and two washes, FITC-rabbit anti-human C1q antibody (Abcam) was applied for 30 min at 37°C. Finally, the bacteria was washed and resuspended in 500 µl PBS for flow cytometry analysis by FACSCalibur. Data from three independent experiments in duplicate were analyzed by Flow Jo 7.6.1 software (33). The assay was repeated three times.

### Hemolytic Assay

The hemolytic activity of SntA protein was measured as previous study (33). For the classical pathway, sheep red blood cells (SRBCs) (Baiji, Zhengzhou, China) were washed with pre-cooled DGHB^++^ buffer for three times and diluted to a concentration of 1 × 10^9^ cells/ml. Then the SRBCs solution was mixed with equal volume of ambocepter (1:1,000; Baiji) and rotated at 37°C for 20 min. After two washes, 0.2% NHS was incubated with 0–100 µg/ml SntA at 37°C for 15 min, then the mixtures were incubated with 3 × 10^8^ cells/ml SRBCs at 37°C for 1 h. BSA was used as a negative and the total volume is 150 µl. After centrifugation at 800 *g*, the hemolytic activity was calculated by spectrophotometric measurement of absorbance at 405 nm. For the alternative pathway, rabbit red blood cells (RRBCs) (Baiji) were washed with pre-cooled Mg^2+^-EGTA-DGHB buffer (4.2 mM Hepes, pH 7.4, 59 mM NaCl, 2.08% glucose, 0.08% gelatin, 7 mM MgCl_2_, 10 mM EGTA) for three times and diluted to a concentration of 1 × 10^9^ cells/ml. 1.25% NHS was incubated with 0–100 µg/ml SntA at 37°C for 15 min, then the mixtures were incubated with 3 × 10^8^ cells/ml RRBCs at 37°C for 1 h. The hemolytic activity of alternative pathway was determined as the classical pathway. These data are presented as mean ± SD from three separate experiments.

### Complement Activation Assay

To detect whether SntA could activate complement directly, complement activation ELISA were performed as described previously (33). 2 µg/ml SntA, 2 µg/ml BSA, 10 µg/ml IgM (Berseebio, Beijing, China) for the classical pathway, 100 µg/ml Mannan (Sigma) for the lection pathway, and 20 µg/ml Zymosan (Sigma) for the alternative pathway were coated to 96-well plates in PBS. The plates were washed with PBS supplemented with 0.05% Tween20 for each step. After blocking with the blocking buffer, 0–7% NHS in DGHB^++^ buffer was applied for classical pathway, 0–7% depleted of protein C1q serum (Quidel) in DGHB^++^ buffer was applied for lectin pathway, and 0–7% NHS in Mg^2+^-EGTA-DGHB buffer was applied for alternative pathway. After incubation for 1 h, the plates were washed and C3b rabbit polyclonal antibody (1:1,000; Proteintech, Wuhan, China) was poured to plates to detect C3 deposition at 37°C for 1 h. All the ELISA assays, the HRP-conjugate goat-anti-rabbit IgG was used as the second antibody at 37°C for 1 h before chromogenic reaction. These data are presented as mean ± SD from three separate experiments.

## Results

### SntA Contributes to the Virulence of *S. suis*

**Figure 1:**
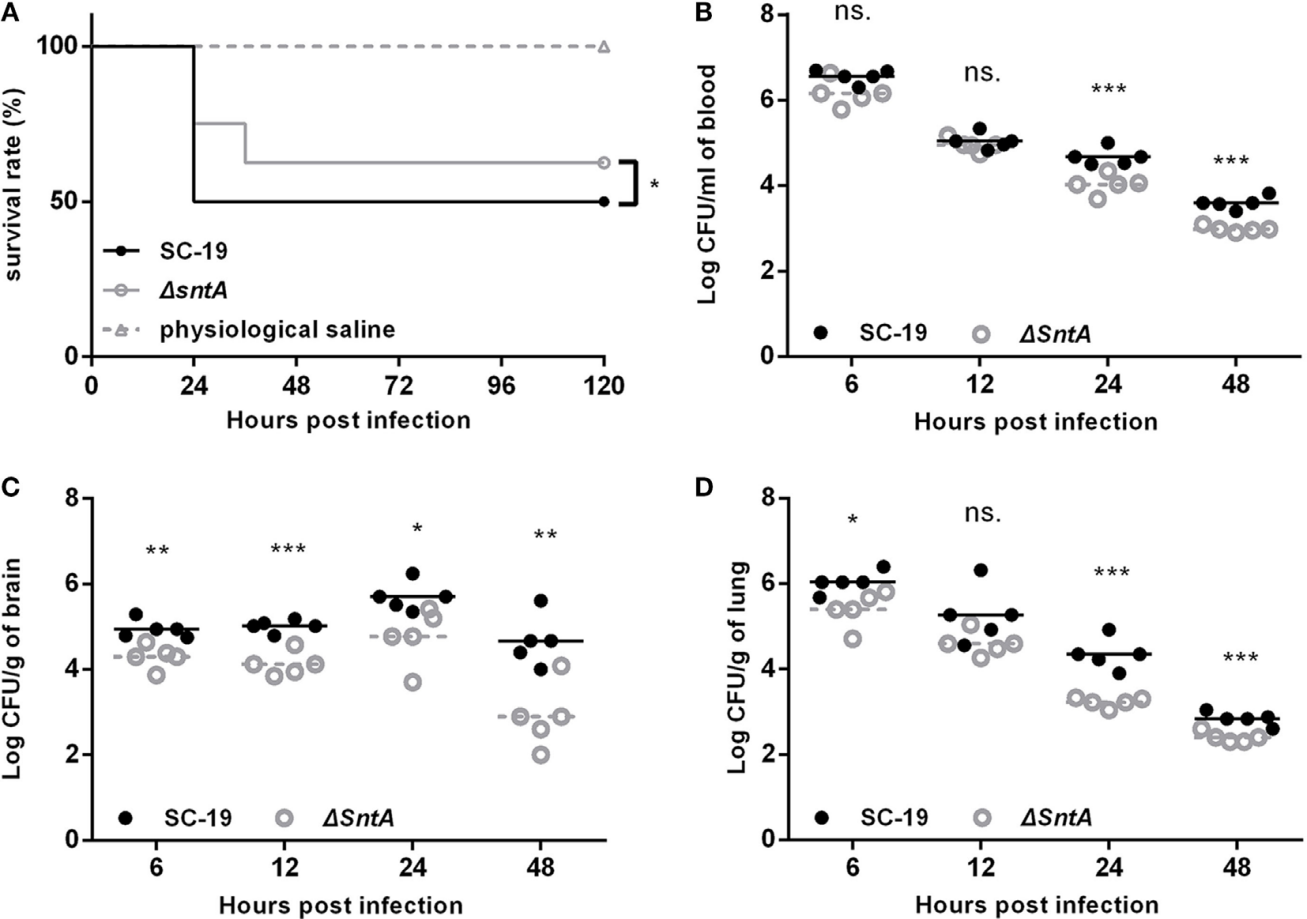
SntA contributes to pathogenesis in mouse infection model. **(A)** Survival curves for mice challenged with *Streptococcus suis*. Groups of eight mice were injected separately and intraperitoneally with 2 × 10^9^ colony forming units (CFU)/mouse of SC-19 and Δ*sntA*. Eight mice inoculated with physiological saline were served as a negative control. Significant difference in survival between different groups was analyzed by Log Rank test for trend. Bacterial loads in blood **(B)**, brain **(C)**, and lung **(D)** were shown. Seven mice per group were intraperitoneally infected with the mixtures of SC-19 and Δ*sntA* at ratio 1:1 (dose of 5 × 10^8^ CFU/mouse). Results were shown as log_10_ of recovered bacteria counts after deletion of the highest and lowest value (CFU/ml in blood and CFU/g in organs). The statistical significance was shown by asterisks (unpaired *t* test; ***p < 0.001; **p < 0.01; *p < 0.05; ns, *p* > 0.05).

Two mouse infection models were performed to investigate the contribution of *sntA* on virulence. The growth curves of *S. suis* presented by OD and CFU were measured before infection in mice. Results showed that no significant differences were observed in SC-19, Δ*sntA*, and CΔ*sntA* in both OD and CFU (Figure S2 in Supplementary Material). First, groups of eight mice were intraperitoneally infected with 2 × 10^9^ CFU/mouse SC-19 and Δ*sntA*. Physiological saline was used as a negative control. Survival rate of infected mice was measured within 5 days post infection (dpi). We observed that four of mice infected with SC-19 died within 24 hpi, another four showed obvious limping, shivering, and central nervous system failure within 5 dpi. By contrast, only two mice infected with Δ*sntA* died within 24 hpi, one mouse died within 36 hpi, and another five mice were survived during the observation within 5 dpi (Figure 1A). This revealed that significant difference was observed in survival rate of SC-19 and Δ*sntA* infected mice. Second, groups of seven mice were intraperitoneally infected with the mixtures of SC-19 and Δ*sntA* at the ratio 1:1 (5 × 10^8^ CFU/mouse). Blood and brain, lung colonization were recovered at 6, 12, 24, and 48 hpi, respectively. The efficiency of colonization of SC-19 was much higher at 24 and 48 hpi than that of Δ*sntA* in blood (Figure 1B) and lung (Figure 1D). In addition, significant differences were observed in brain from 6 to 48 hpi (Figure 1C). The results showed that SntA contributed to the survival in blood and the colonization in specific organs.

### SntA Possess Obvious Anti-Phagocytic Activity

**Figure 2:**
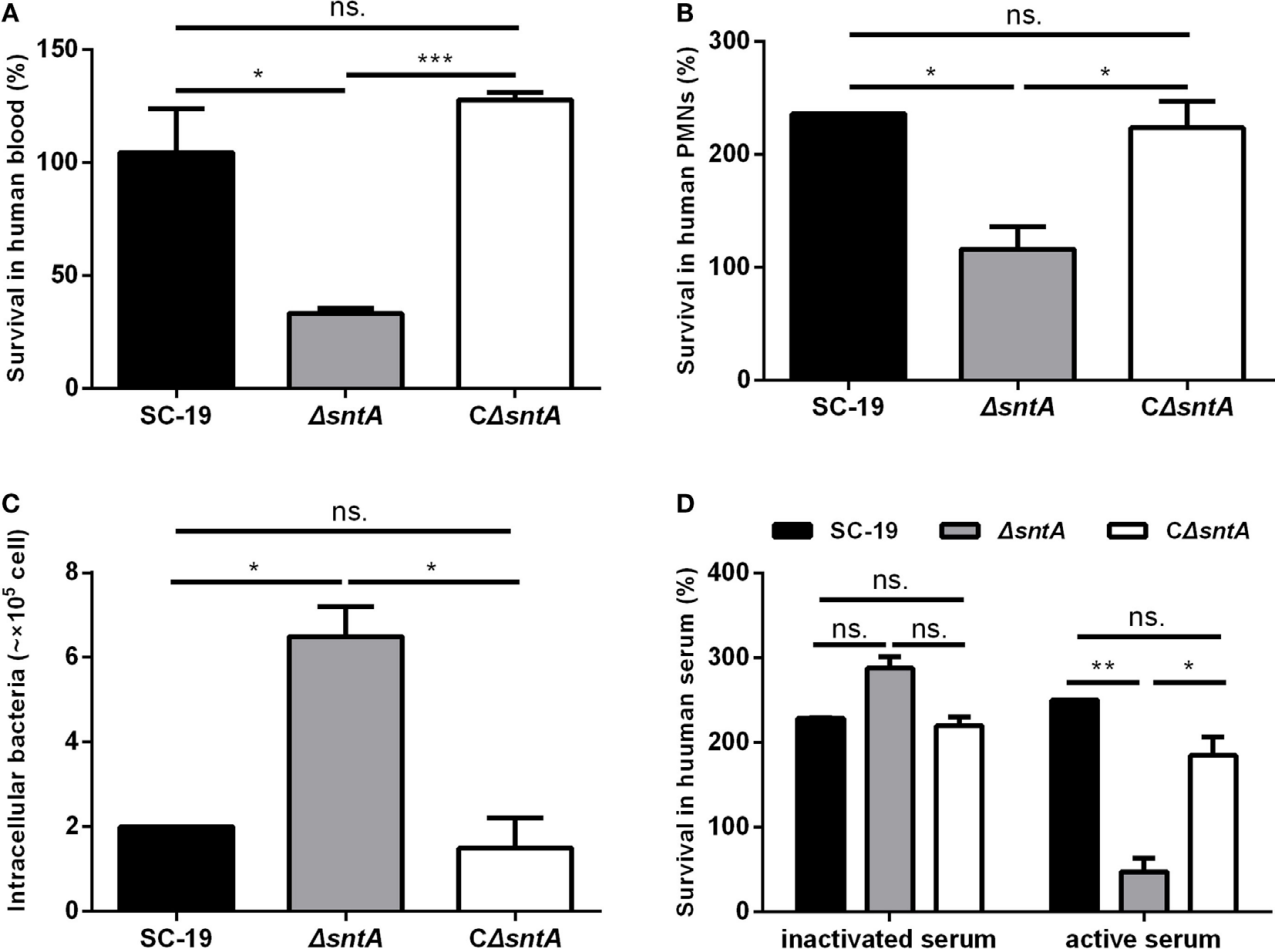
SntA possess anti-phagocytic activity. **(A)** Survival rate of *Streptococcus suis* in human blood. **(B)** Survival rate of *S. suis* in PMNs separated from freshly human blood. **(C)** The numbers of *S. suis* phagocytized in mouse macrophage RAW264.7 cells. **(D)** Survival rate of *S. suis* in human active and inactivated serum. Results were expressed from three independent experiments performed in triplicate. The statistical significance was showed by asterisks (unpaired *t* test; ***p < 0.001; **p < 0.01; *p < 0.05; ns, *p* > 0.05).

We constructed a complementary strain of *sntA*, as confirmed by genome PCR (Figure S1A in Supplementary Material) and RT-PCR (Figure S1B in Supplementary Material). To investigate the anti-phagocytosis mediated by SntA, three bacteria killing assays and one phagocytosis assay were performed. In blood killing assay, the survival rate of SC-19 in human blood was 105%, but the absence of *sntA* decreased the survival rate to only 33% (Figure 2A). Similar results were observed in mouse blood killing assay (Figure S3A in Supplementary Material). In PMNs killing assay, PMNs supplemented with 20% active human serum were used to test the survival abilities of *S. suis* in human neutrophils. Results revealed that the survival rate of Δ*sntA* was significantly decreased compared to SC-19 and CΔ*sntA* (Figure 2B). In phagocytosis assay, RAW 264.7 cells were used to test anti-phagocytic activity of *S. suis*. Results demonstrated that the phagocytic numbers of Δ*sntA* were much higher comparing with SC-19 and CΔ*sntA* (Figure 2C). In the human serum killing assay, the active and inactivated human serum were used to investigate the role of complement on *S. suis* clearance. For the *sntA*-deleted mutant strain, only 48% viable Δ*sntA* bacteria survived in freshly active human serum. The presence of *sntA* significantly increased the survival rate of *S. suis* up to 250% (SC-19) and 185% (CΔ*sntA*), respectively. No obvious differences were observed in the survival rates of *S. suis* in inactivated serum (Figure 2D). Normal mouse serum was also used in this assay, and similar results were obtained (Figure S3B in Supplementary Material). No significant differences were observed between SC-19 and CΔ*sntA* in all tests. These data demonstrated that SntA was involved in resistance to phagocytosis and the bactericidal activity of blood, PMNs, and NHS dependent on complement. This reveled that SntA could obviously contribute to anti-phagocytosis and this may be involved in complement.

### SntA Inhibits the C3 Deposition and MAC Formation on *S. suis*

**Figure 3:**
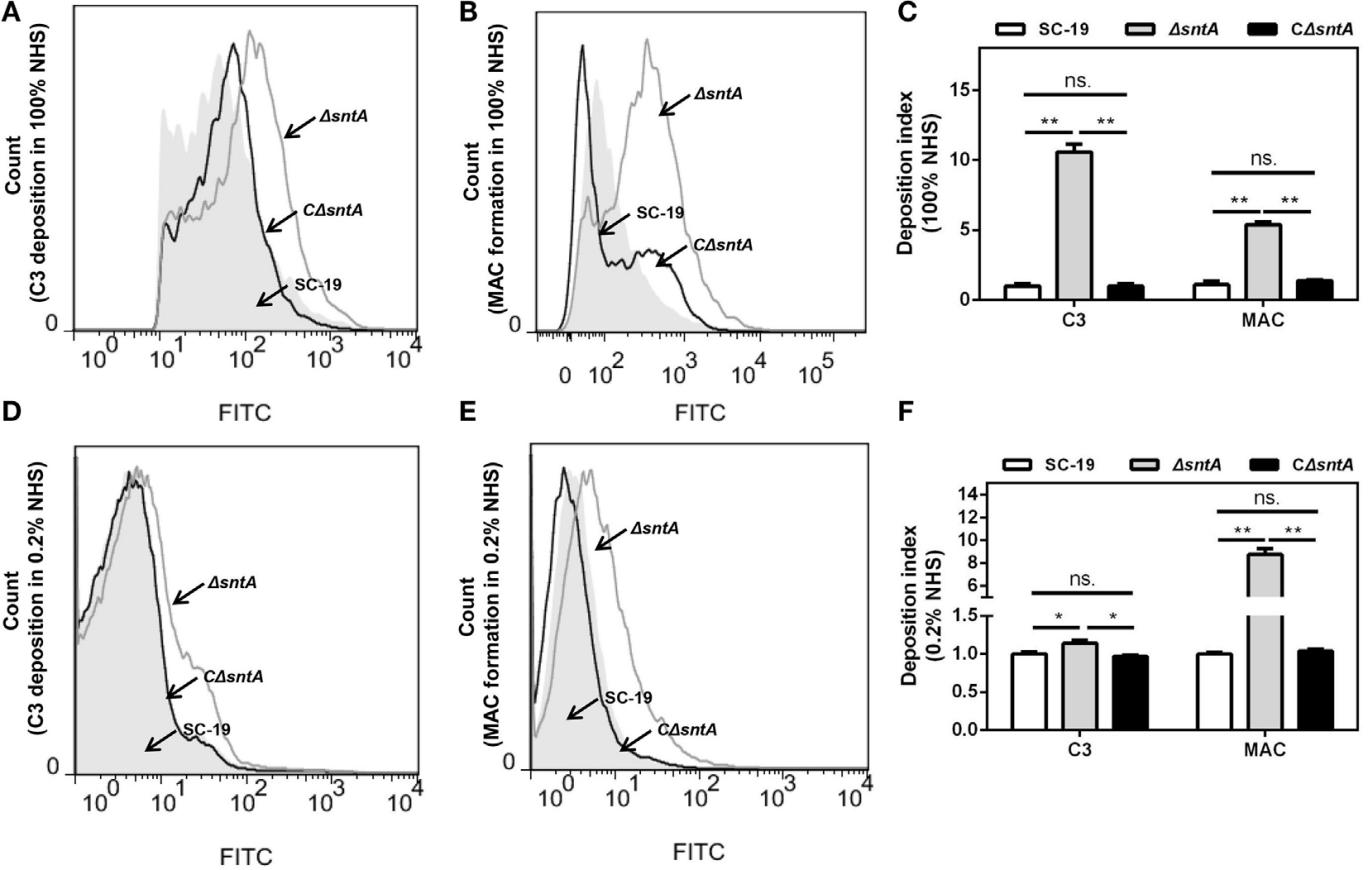
SntA inhibits C3 deposition and membrane attack complex (MAC) formation. **(A–F)** The C3 deposition and MAC formation capacities were presented by count of positive cell aggregated with fluorescence in the presence of complement (100 and 0.2% normal human serum). The representative histogram of flow cytometry from three independent experiments was exhibited **(A,B,D,E)**. Geometric mean fluorescence intensity (GMFI) value was measured from three independent experiments performed in duplicate. The GMFI value of C3 deposition and MAC formation of SC-19 in all tests was set as 1.00, the corresponding Δ*sntA* and CΔ*sntA* values were proportional achieved. The resulting C3 deposition and MAC formation index were presented **(C,F)**. The statistical significance was calculated by two-way ANOVA of Sidak’s correct for multiple comparison and shown by asterisks (**p < 0.01; *p < 0.05; ns, *p* > 0.05).

SntA possessed anti-phagocytosis, but the effect of SntA on complement activation on *S. suis* was still unclear. To investigate that, the C3 deposition and MAC formation assays were performed in both undiluted and 1:500 diluted human serum. Results showed that the viable bacteria number aggregated fluorescence of Δ*sntA* in C3 deposition was obviously increased compared to SC-19 and CΔ*sntA* in undiluted human serum (Figures 3A,C). The efficiency of MAC formation was significantly higher comparing with SC-19 and CΔ*sntA* (Figures 3B,C) as well. To assess the effect of SntA on complement activation through the classical pathway, 1/500 NHS was used. Results revealed that C3 deposition and MAC formation on surface of Δ*sntA* was significantly higher compared with SC-19 and CΔ*sntA* in 1/500 diluted human serum (Figures 3D–F). No significant differences were observed between SC-19 and CΔ*sntA* in all tests. These assays demonstrated that SntA could inhibit the complement pathway.

### SntA Is a Cell Wall Anchored Protein

**Figure 4:**
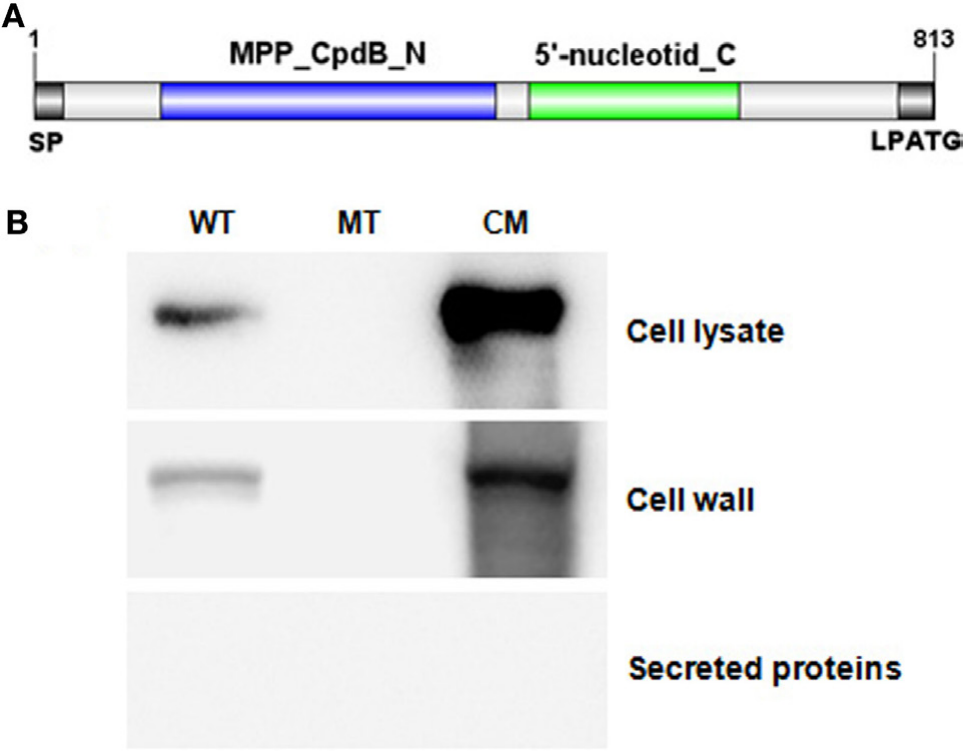
SntA was a conserved cell wall anchored protein. **(A)** Conserved domain of SntA was predicted in NCBI database (https://www.ncbi.nlm.nih.gov/cdd). SntA had two conserved domain: MPP_CpdB_N and 5′-nucleotid_C, and a cell wall attached LPATG motif. **(B)** Western-blot was used to determine surface location of SntA. Cell lysate, cell wall proteins, and secreted proteins were ran 10% SDS-PAGE, transferred onto PVDF membrane and detected by mouse anti-SntA poly-antibody following by HRP-conjugate goat-anti-mouse IgG. WT, SC-19; MT, Δ*sntA*; CM, CΔ*sntA*.

The cell wall attached protein LPXTG motif region was found at C terminal of amino acid sequence of SntA (Figure 4A). To confirm this prediction, western-blot analysis was performed. Results showed that SntA expressed in cell lysate extracted from SC-19 and CΔ*sntA*, but not detected in Δ*sntA*. Cell wall proteins extracted by mutanolysin and secreted proteins sedimentated by TCA-acetone were used to further determine the location of SntA in *S. suis*. SntA was demonstrated to express in cell wall proteins, and not in secreted proteins (Figure 4B). These assays indicated that SntA existed in cell wall.

### SntA Interacts With Complement C1q

**Figure 5:**
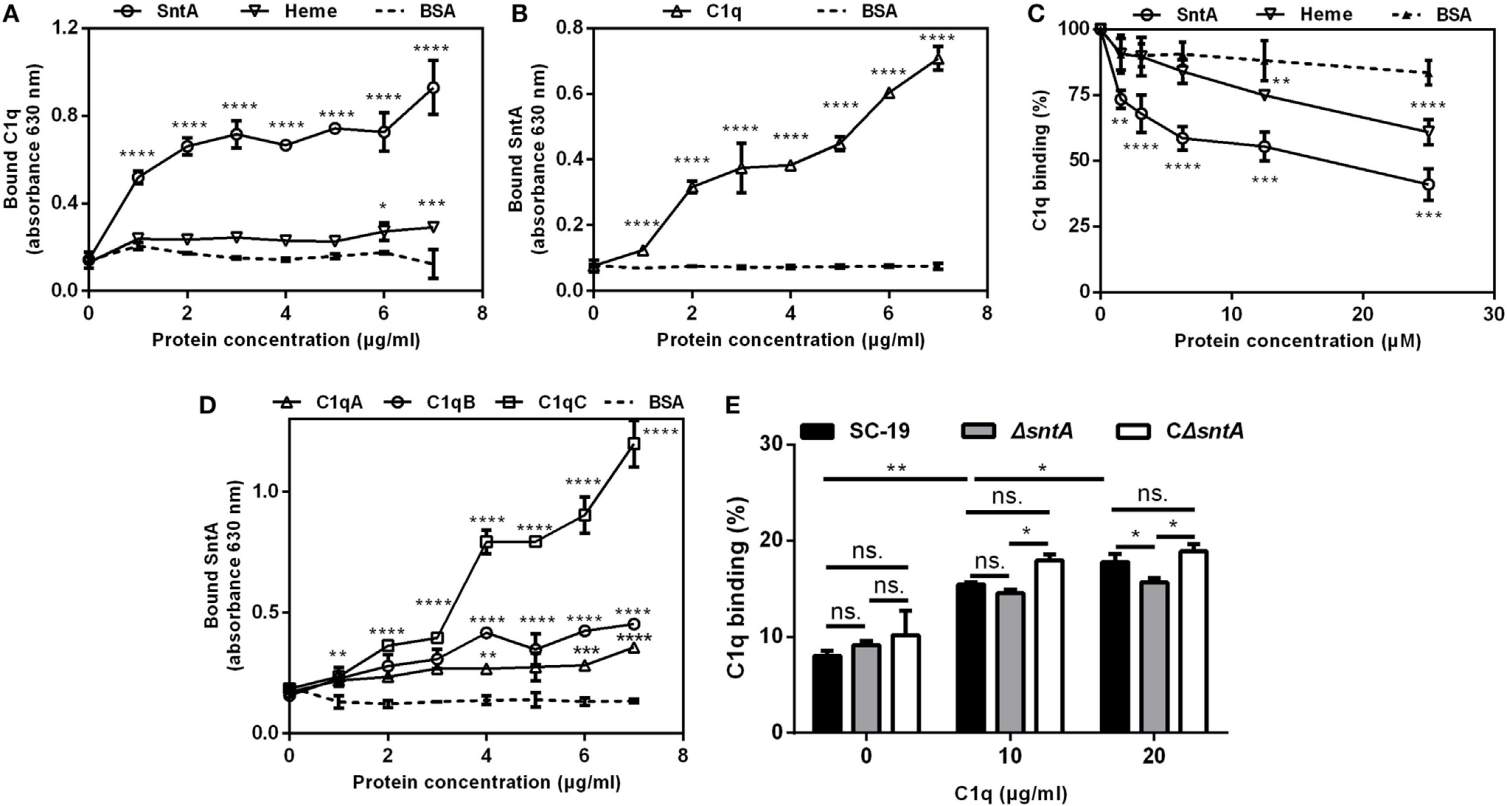
SntA interacts with C1q. **(A)** C1q was applied to SntA-, heme-, and BSA-coated plates to test the C1q bound capacity. **(B)** SntA was applied to C1q- and BSA-coated plates to test the SntA bound capacity. **(C)** SntA, heme, or BSA was premixed with C1q and applied to IgG-coated plates to test the binding capacity between C1q and IgG, which indicating the binding capacity between SntA and C1q. **(D)** SntA was applied to C1qA-, C1qB-, C1qC-, and BSA-coated plates to test the SntA bound capacity. **(E)** Increasing concentrations of C1q were incubated with *Streptococcus suis* to detect the binding capacity by FITC-anti-human C1q antibody. Results were expressed from three independent experiments performed in triplicate. The statistical significance was calculated by two-way ANOVA of Sidak’s correct for multiple comparison and shown by asterisks (****p < 0.0001; ***p < 0.001; **p < 0.01; *p < 0.05; ns, *p* > 0.05).

To assess the binding capacity between SntA and C1q, the direct and indirect bound assays were performed. In direct bound assay, C1q could significantly bind with SntA in a dose-dependent manner in both C1q-coated and SntA-coated plates (Figures 5A,B). The known C1q-binding molecule heme was used as a positive control and BSA as a negative control. In inhibition assay, both SntA and heme could significantly inhibit the binding capacity between plasma purified C1q and aggregated human IgG on IgG-coated plates, and the inhibitory effect of SntA was much stronger than the positive control heme (Figure 5C). Furthermore, three subunits of C1q including C1qA, C1qB, and C1qC could obviously interact with SntA in a dose-dependent manner as well (Figure 5D). These results indicated that recombinant SntA could bind to C1q. To investigate whether SntA bound with C1q on bacterial surface, flow cytometry was performed. A dose-dependent increase of C1q-binding capacity was observed on *S. suis*. Compared with SC-19 and CΔ*sntA*, a significant reduction of C1q-binding capacity was observed in Δ*sntA* when 20 µg/ml C1q was added (Figure 5E). Taken together, SntA could interact with C1q and act as a C1q ligand on bacterial surface.

### SntA Competitively Bind With C1q

**Figure 6:**
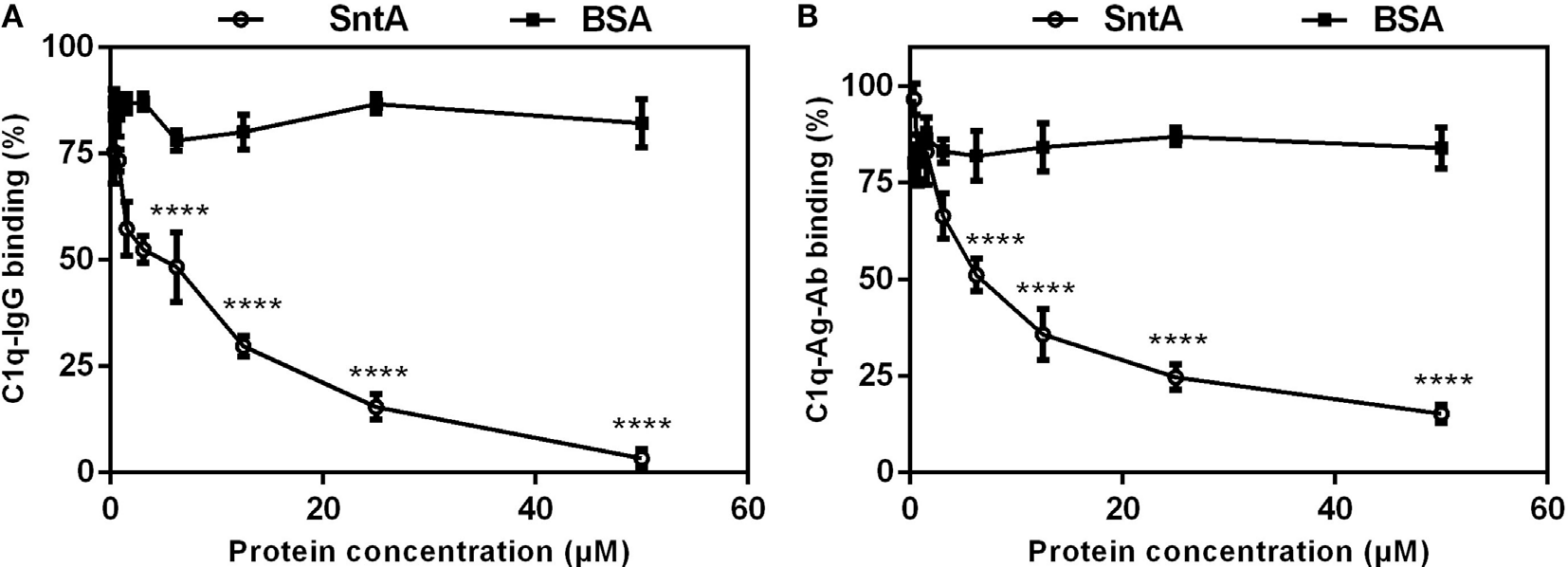
SntA competitively binds with C1q. **(A)** Inhibition of IgG–C1q binding. Increasing concentrations of SntA were premixed with C1q and applied to IgG-coated plates to test the inhibitory capacity of SntA to C1q–IgG binding. **(B)** Inhibition of C1q–Ag–Ab binding. IgG was applied to tetanus toxoid-coated plates for IgG-containing immune complex formation. Increasing concentrations of SntA were premixed with C1q and added to plates to test the inhibitory capacity of SntA to C1q–Ag–Ab binding. BSA was served as a negative control. Results were expressed from three independent experiments performed in triplicate. The statistical significance was calculated by two-way ANOVA of Sidak’s correct for multiple comparison and shown by asterisks (****p < 0.0001; ns, *p* > 0.05).

The classical pathway is triggered through the recognition of antigen-antibody complex on micro surface by recognition molecule C1q (4). To assess the effect of C1q-binding protein SntA on the recognition step of the classical complement pathway, competitive binding assays were performed. In C1q–IgG binding assay, result showed that SntA strongly decreased the C1q–IgG binding capacity, and 50 µM SntA inhibited the binding capacity down to 3.34% (Figure 6A). In C1q–Ag–Ab binding assay, the binding capacity was significantly decreased in a dose-dependent manner and 84.7% of it was remarkably inhibited with 50 µM SntA (Figure 6B). Results demonstrated that SntA could inhibit C1q–IgG binding and C1q–Ag–Ab binding by competitive interaction with C1q.

### SntA Inhibits Hemolytic Activity

**Figure 7:**
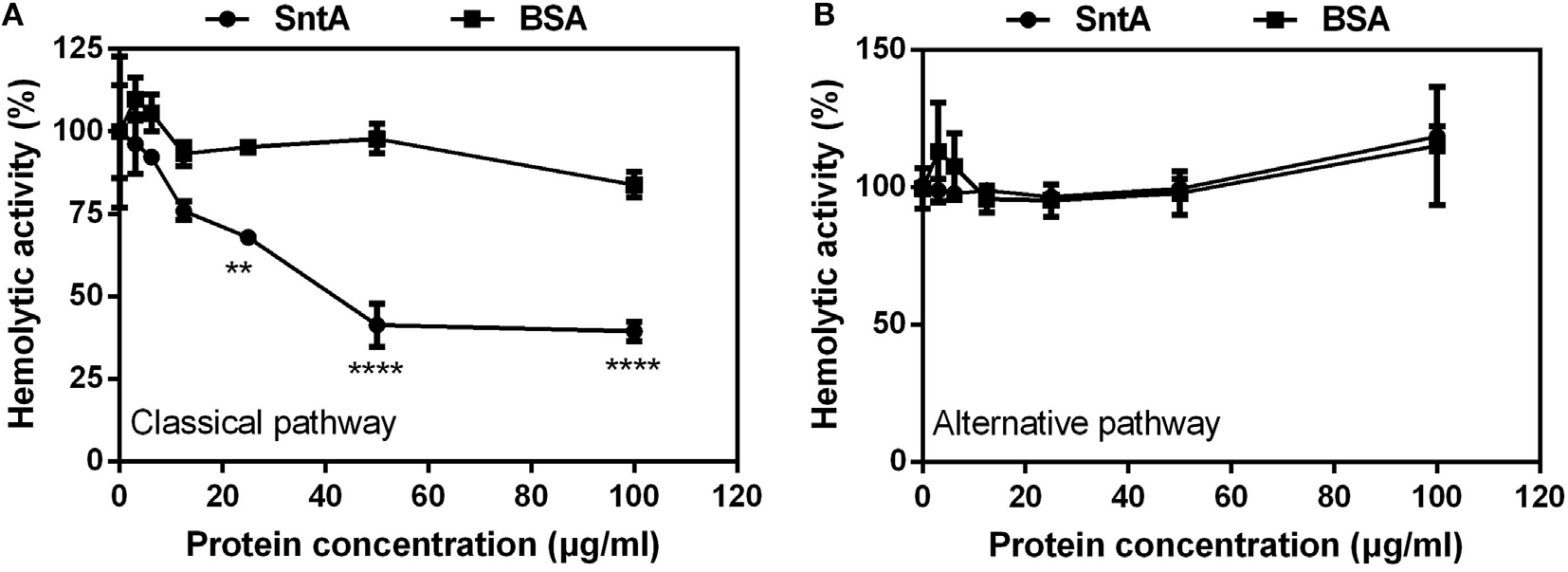
SntA inhibits hemolytic activity. **(A)** To measure the inhibitory effect of SntA on the classical pathway, antibody-coated sheep erythrocytes were subjected to complement attack from normal human serum (NHS) in the presence of the increasing concentrations of SntA. BSA was used as a negative control. The degree of lysis was estimated by measurement of the release of hemoglobin. **(B)** To measure the inhibitory effect of SntA on the alternative pathway, rabbit erythrocytes were subjected to complement attack from NHS in the presence of the increasing concentrations of SntA. BSA was used as a negative control. Cell lysis was measured like **(A)**. The absorbance without inhibitor was set to 100%. Results were expressed from three independent experiments performed in triplicate. The statistical significance was calculated by two-way ANOVA of Sidak’s correct for multiple comparison and shown by asterisks (****p < 0.0001; **p < 0.01; ns, *p* > 0.05).

SntA inhibited complement pathway, in which pathway SntA acted remain unclear. Hemolytic activity assay is often used to assess the activity of the classical pathway of complement activation with sheep erythrocytes (SRBCs) and of alternative pathway with rabbit erythrocytes (RRBCs) (33). So, to test in which pathway SntA inhibited complement, the hemolytic activity assays were performed. Results showed that SntA remarkably inhibited the hemolytic activity of SRBCs in a dose-dependent manner mediated by the classical pathway in the presence of NHS (Figure 7A). No inhibitory effect was observed in the negative control of BSA. However, SntA did not inhibit the hemolytic activity of RRBCs mediated by the alternative pathway (Figure 7B). These data revealed that SntA could inhibit the hemolytic activity mediated by the complement classical pathway.

### SntA Directly Activates Complement Pathways

**Figure 8:**
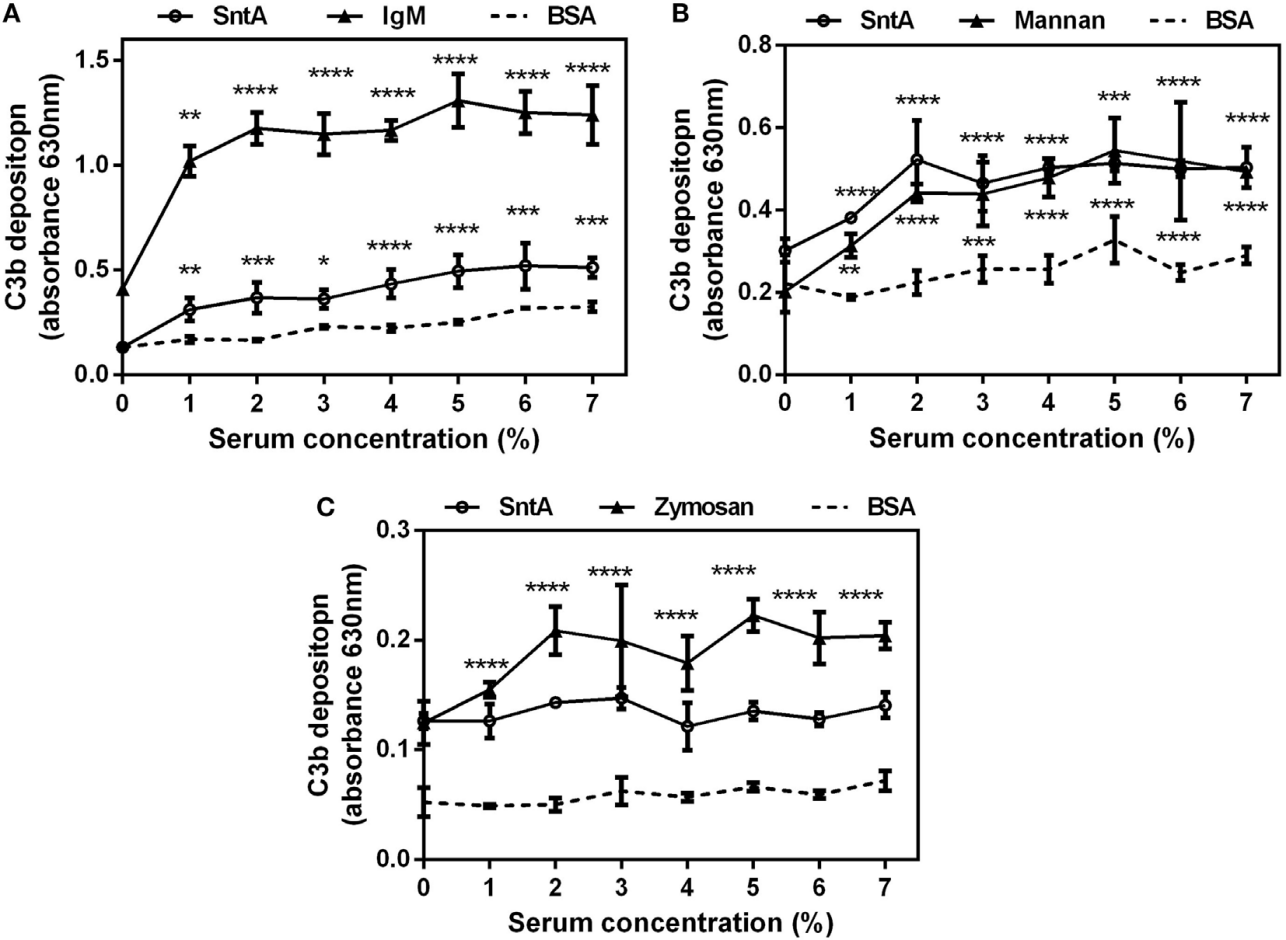
SntA can directly activate the classical and lectin complement pathways. **(A)** Normal human serum (NHS) in DGHB^++^ buffer was applied to the plates pre-coated with SntA, IgM was acted as a positive control and BSA as a negative control to detect C3b deposition. **(B)** Human serum depleted of C1q in DGHB^++^ buffer was applied to the plates pre-coated with SntA, Mannan as a positive control to detect C3b deposition. **(C)** NHS in Mg^2+^-EGTA-DGHB buffer was applied to the plates pre-coated with SntA, Zymosan as a positive control to detect C3b deposition. Results were expressed from three independent experiments performed in triplicate. The statistical significance was calculated by two-way ANOVA of Sidak’s correct for multiple comparison and shown by asterisks (****p < 0.0001; ***p < 0.001; **p < 0.01; *p < 0.05; ns, *p* > 0.05).

To test whether SntA could activate complement directly, the complement activation assays were performed. In the classical pathway, the increasing concentrations of 0–7% NHS were applied to the SntA pre-coated plates to detect C3b deposition with specific C3b polyclonal antibody. IgM acted as a positive control and BSA as a negative control. Significant activation was observed on the SntA pre-coated plates and not on the BSA pre-coated plates (Figure 8A). This showed that SntA could activate complement through the classical pathway, although the activation level was lower than the positive control. In the lectin pathway, microtiter plates pre-coated with SntA and positive control Mannan exhibited strong complement activation mediated by the lectin pathway when human serum depleted of C1q was used (Figure 8B). Result demonstrated that SntA could activate the lectin pathway directly. In the alternative pathway, NHS in Mg^2+^-EGTA-DGHB buffer was used to block classical activation pathway and detect complement activation mediated by the alternative pathway. Result showed that no significant activation was observed in the plates pre-coated with SntA, while the positive control Zymosan obviously activated the alternative pathway (Figure 8C). Thus, SntA could directly activate the complement by the classical and lectin pathway, not the alternative pathway.

## Discussion

Streptococcal toxic shock like syndrome, meningitis, and septicemia are the most important characteristics of the recently emerging *S. suis* infection, especially in Southeast Asia. During infection, the pathogen must compete with the normal microflora, resist defense mechanisms of the local mucosal immunity, adhere and invade the mucosal epithelial cell barrier, subsequently reach and survive in the blood stream, and finally invade multiple organs including spleen, liver, kidney, and lung, even entry BBB (36). A plenty of factors have been demonstrated to be associated with these processes [reviewed in Ref. (37)]. However, how *S. suis* escape the immune surveillance to invade into blood and most organs is largely unknown. In this study, we found that the surface heme-binding protein SntA of *S. suis* (24) contributes to the resistance of C3 deposition and MAC formation on bacterial surface, the abilities of anti-phagocytosis, survival in blood, and *in vivo* colonization. SntA can interact with C1q, and inhibit the hemolytic activity *via* the classical pathway. SntA could also directly activate classical and lectin pathways, resulting in complement consumption. These two complement evasion strategies may be crucial for the pathogenesis of the zoonotic pathogen *S. suis*.

In our previous study, SntA is identified as a heme-binding protein which can interact with the host antioxidant protein AOP2 and inhibit its antioxidant activity, and thus contribute to the survival and pathogenesis of *S. suis* in pigs (24). This protein is a bifunctional 2′,3′-cyclic nucleotide 2′-phosphodiesterase/3′-nucleotidase, which is distributed in many species of Gram-positive bacteria (24). In the present study, mouse infection model is used to further study the function of SntA involving in the pathogenesis. Results show that SntA facilitates in *S. suis* survival in blood (Figure 1B) and subsequent colonization in some specific organs (Figures 1C,D). To entry the bloodstream and spread to distant organs, the Gram-positive pathogens usually utilize opsonophagocytic clearance function of neutrophils and macrophages (38). SntA is demonstrated to contribute to the anti-phagocytosis of *S. suis* mediated by neutrophils and macrophages and the survival in whole blood and serum *in vitro* (Figure 2). The efficient phagocytosis is an important innate immune mechanism for controlling infection of extracellular bacteria such as *Streptococci*, and this process requires serum with intact complement activity (39). Recently, many complement inhibitors target at complements such as FH (29), C1q (23, 40), C3 (41), C4BP (42), mediate complement evasion, and subsequently contribute to pathogenesis in Gram-positive bacterium. In order to investigate whether SntA involves in the complement evasion of *S. suis*, a series of experimentations are carried out to this end.

First, the complement deposition assays are performed. The C3 deposition and MAC formation on the surface of the mutant strain Δ*sntA* are significantly higher than those on the parental strain SC-19 and complementary strain CΔ*sntA* (Figure 3). This indicates that SntA involved in complement activation by *S. suis*. The undiluted and 1:500 diluted human serum (NHS) are used in the complement deposition assays, respectively, C3 deposition and MAC formation on the surface of Δ*sntA* are always significantly higher than those on SC-19 and CΔ*sntA* (Figure 3). This suggests that SntA could inhibit both classical pathway and alternative pathway because it has been documented that the classical pathway is activated by using diluted NHS (35), whereas alternative pathway is probably activated by using undiluted NHS in the complement activation assays (30).

Hemolytic assay is often used to assess the activation of the classical pathway by using sheep erythrocytes and the activation of alternative pathway by using rabbit erythrocytes (33). To confirm which complement pathway is inhibited by SntA, hemolytic assays have been performed using recombinant SntA protein. The results show that SntA could inhibit the hemolytic activity of SRBCs, but not of rabbit erythrocytes (Figure 7). This indicates that SntA could only inhibit the classical pathway. Furthermore, SntA is confirmed as a cell wall anchored protein that could interact with C1q in a dose-dependent manner (Figures 4 and  5). ELISA experiments also suggest that SntA could interact with the three C1q subunits C1qA, C1qB, and C1qC, respectively (Figure 5). It is very interesting whether this interaction results in the complement evasion. To this end, the competitive binding assays have been done by ELISAs reported previously (35). The results show that SntA could competitively bind C1q with IgG and Ag–Ab complex (Figure 6). Thus, SntA can interact with C1q and inhibit the hemolytic activity mediated by the classical pathway, consequently contribute to complement evasion. Similar inhibitory mechanism has been identified in other Gram-positive strain. *Streptococcus pneumococcal* (33) and *S. aureus* (23) infection. These bacterial proteins interact with C1q and specially inhibit the classical complement pathway, consequently facilitate *pneumococcal* complement escape (23, 33).

Concerning that C1q is a complex of 18 polypeptide chains consisting of six C1qA, C1qB, and C1qC subunits, the binding assays using each subunit could not properly reveal the real interactions between SntA and C1q although it has been reported that the globular heads of the recombinant subunits can assemble to form the high molecular weight oligomers (43). Interestingly, heme has also been demonstrated to inhibit the classical pathway through binding with C1q (35), and SntA is a heme-containing protein (24), it warrants further investigation whether the heme in SntA or SntA as a hemeprotein involves in the complement evasion. To this end, we are trying to obtain the recombinant SntA without heme and holo-form SntA although this is not an easy thing.

To look at whether SntA can directly activate complement pathways, the recombinant SntA protein is subjected to activate the complement C3. The results show that SntA could directly activate both classical and lectin pathways but not alternative pathway (Figure 8). This indicates that SntA could mediate complement evasion through complement consumption as well (33). In *S. pneumococcal*, PepO can activate complement through the classical and alternative pathway (33), while anther complement inhibitor phosphoglycerate kinase cannot activate complement (44). Research about complement evasion mediated by complement consumption is not much. The mechanism of this process warrants further study.

In conclusion, *S. suis* cell wall anchored heme-binding protein SntA mediates complement evasion by inhibition of complement activation and complement consumption. The inhibition of complement activation may be *via* the SntA–C1q interaction. These two complement evasion strategies may be crucial for the pathogenesis of *S. suis*.

## Ethics Statement

Animal experiments were approved by the Laboratory Animal Monitoring Committee of Huazhong Agricultural University and performed strictly according to the recommendations in the Guide for the Care and Use of Laboratory Animals of Hubei Province, China. Venous blood samples were provided by healthy donors and collected in accordance with the approved guidelines. Approval was obtained from the Institutional Medical Ethics Committee of Huazhong Agricultural University and the healthy donors provided written informed consent in accordance with the Declaration of Helsinki.

## Author Contributions

The experiments were performed mainly by SD, some experiment material and data were provided by TX and LY, and some experiments were performed with the assistance of TX, QF, JZ, LC, and JL. SD analyzed the data. The study was conceived and designed by SD and RZ. SD and RZ wrote the manuscript.

## Conflict of Interest Statement

The authors declare that the research was conducted in the absence of any commercial or financial relationships that could be construed as a potential conflict of interest.

## Supplementary Material

Figure S1 Identification of *Streptococcus suis* strains SC-19, Δ*sntA*, and CΔ*sntA*. The *sntA* gene and its flanked genes (B9H01_10065 and B9H01_10075) were amplified by genome PCR **(A)** and reverse transcription-PCR **(B)** using primers SntA_F/R, 10065_F/R, and 10075_F/R, respectively. WT, wild-type strain SC-19; MT, *sntA* gene mutant strain Δ*sntA*; CM, complementary strain CΔ*sntA*.

Figure S2 The growth abilities of *Streptococcus suis* strains SC-19, Δ*sntA*, and CΔ*sntA*. **(A)** Bacterial cell density was measured by spectrophotometer at 600 nm. **(B)** Bacterial colony forming unit count. Results were expressed from three independent experiments performed in triplicate.

Figure S3 SntA possess anti-phagocytic activity. **(A)** Survival rate of *Streptococcus suis* in mouse blood. **(B)** Survival rate of *S. suis* in mouse active and inactivated serum. Results were expressed from three independent experiments performed in triplicate. The statistical significance was showed by asterisks (unpaired *t* test; ****p* < 0.001; ***p* < 0.01; **p* < 0.05; ns, *p* > 0.05).
