# Supplementary figures and images for: The Surface-Exposed Protein SntA Contributes to Complement Evasion in Zoonotic Streptococcus suis
Source: Front Immunol. 2018 May 16;9:1063. doi: 10.3389/fimmu.2018.01063 (PMC5964162; doi:10.3389/fimmu.2018.01063)

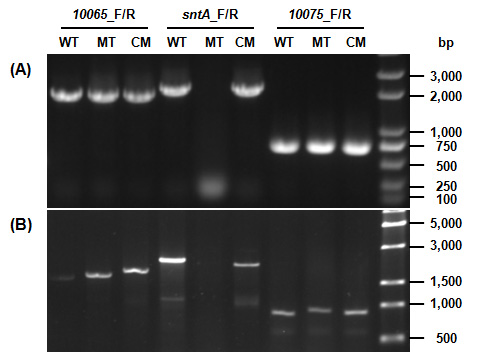

Supplement: Figure S1 — Identification of Streptococcus suis strains SC-19, ΔsntA, and CΔsntA. The sntA gene and its flanked genes (B9H01_10065 and B9H01_10075) were amplified by genome PCR (A) and reverse transcription-PCR (B) using primers SntA_F/R, 10065_F/R, and 10075_F/R, respectively. WT, wild-type strain SC-19; MT, sntA gene mutant strain ΔsntA; CM, complementary strain CΔsntA. [file image_1.jpeg]

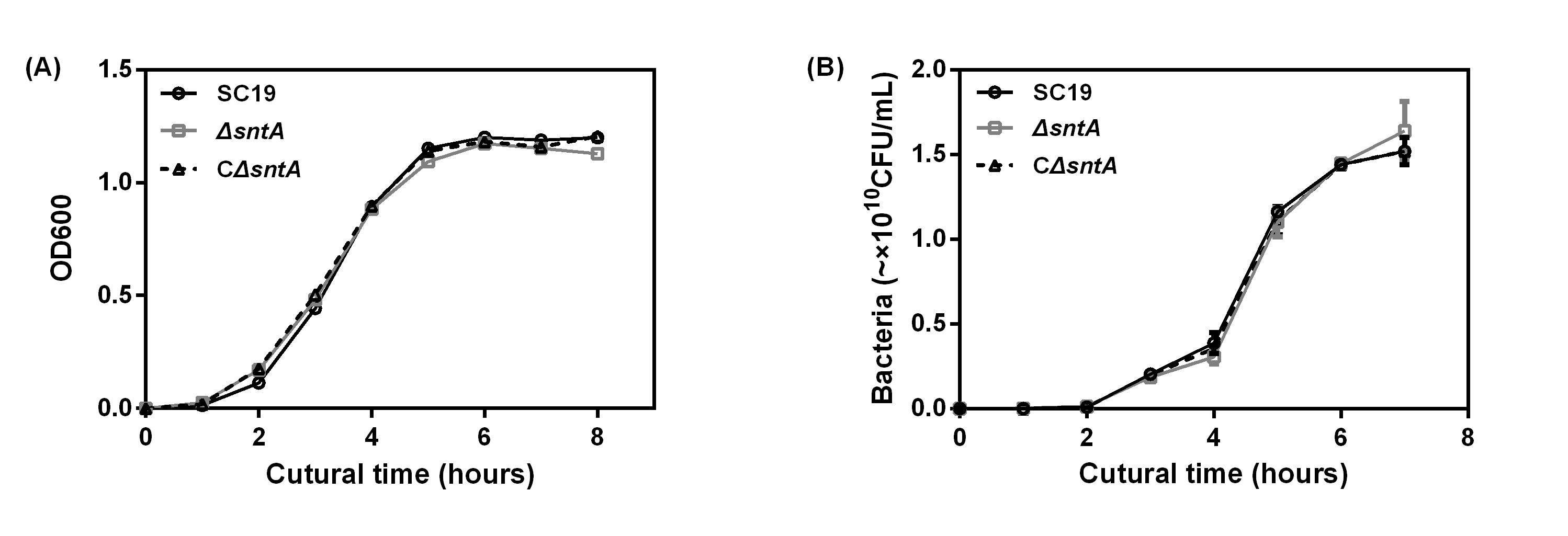

Supplement: Figure S2 — The growth abilities of Streptococcus suis strains SC-19, ΔsntA, and CΔsntA. (A) Bacterial cell density was measured by spectrophotometer at 600 nm. (B) Bacterial colony forming unit count. Results were expressed from three independent experiments performed in triplicate. [file image_2.jpeg]

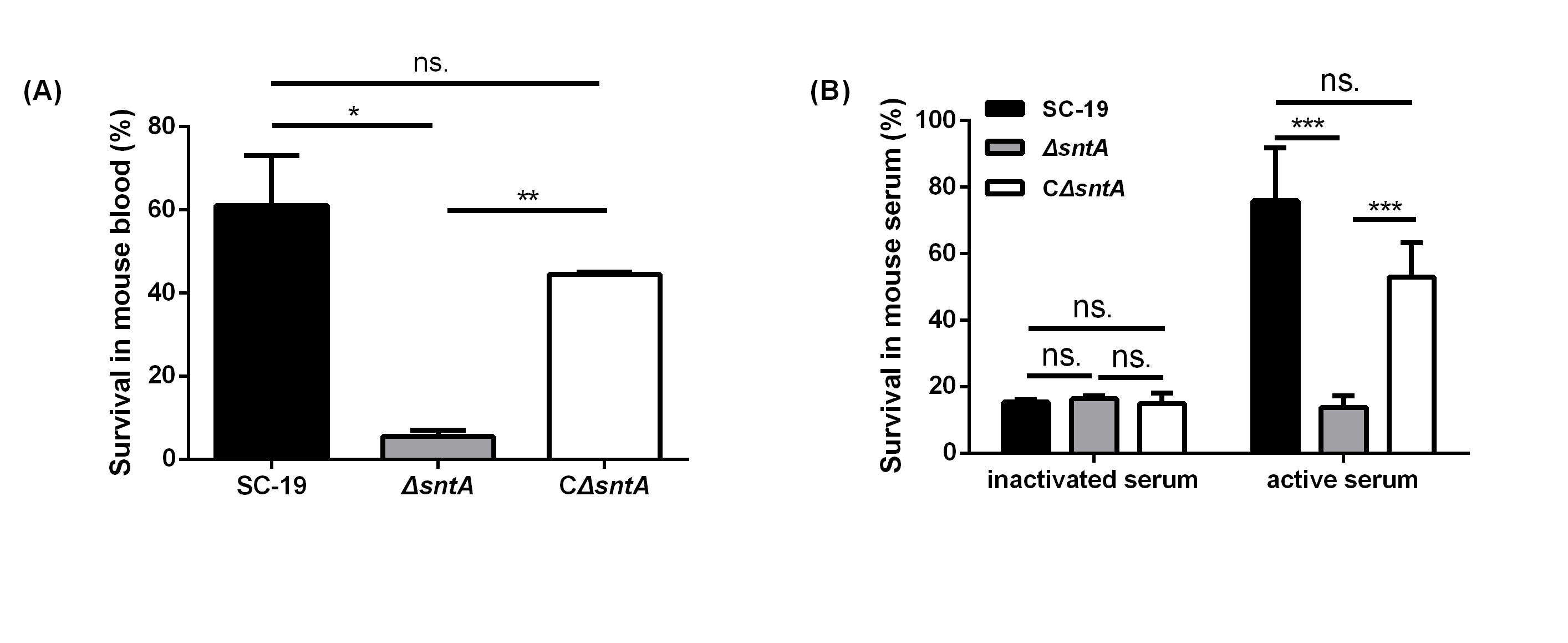

Supplement: Figure S3 — SntA possess anti-phagocytic activity. (A) Survival rate of Streptococcus suis in mouse blood. (B) Survival rate of S. suis in mouse active and inactivated serum. Results were expressed from three independent experiments performed in triplicate. The statistical significance was showed by asterisks (unpaired t test; ***p < 0.001; **p < 0.01; *p < 0.05; ns, p > 0.05). [file image_3.jpeg]
